# Neuroprotective Anesthesia Regimen and Intensive Management for Pediatric Cardiac Surgery with Cardiopulmonary Bypass: a Review and Initial Experience

**DOI:** 10.21470/1678-9741-2016-0064

**Published:** 2017

**Authors:** Jyrson Guilherme Klamt, Walter Villela de Andrade Vicente, Luis Vicente Garcia, Fabio Carmona, João Abrão, Antônio Carlos Menardi, Paulo Henrique Manso

**Affiliations:** 1 Hospital das Clinicas da Faculdade de Medicina de Ribeirão Preto da Universidade de São Paulo (FMRP-USP), Ribeirão Preto, SP, Brazil.

**Keywords:** Pediatrics, Cardiac Surgical Procedures, Neuroprotection, Cardiopulmonary Bypass

## Abstract

This article describes our proposal for routine anesthesia, intraoperative medical management, cerebral and physiological monitoring during pediatric cardiac surgery with cardiopulmonary bypass that intend to provide appropriate anesthesia (analgesia, hypnosis), neuroprotection, adequate cerebral and systemic oxygen supply, and preventing against drugs neurotoxicity. A concise retrospective data is presented.

**Table:** 

Abbreviations, acronyms & symbols	
AEP	= Auditory evoked potentials
BIS	= Bispectral index
CBF	= Cerebral blood flow
CMRO_2_	= Cerebral metabolic rate of oxygen
CPB	= Cardiopulmonary bypass
CT	= Computed tomography
EEG	= Electroencephalogram
MRI	= Magnetic resonance image
NIRS	= Near-infrared spectroscopy technology
NSE	= Neuron specific enolase
PaCO_2_	= Arterial partial pressure of carbon dioxide
rScO_2_	= Cerebral regional oximetry
SaO_2_	= Arterial hemoglobin oxygen saturation
SjO_2_	= Jugular hemoglobin oxygen saturation
SsvcO_2_	= Superior vena cava hemoglobin oxygen saturation

## INTRODUCTION

Perioperative neurologic monitoring of small children has become routine in most major centers and the pertaining data suggest that the technologies identify adverse neurologic events and may indeed predict outcome. Monitoring methods currently used included processed electroencephalography (BIS- bispectral index), cerebral oximetry (NIRS- near-infrared spectroscopy) and Doppler ultrasound. On the other hand, neuroprotective therapies (drugs and procedures) are still controversy and used empirically.

Pediatric cardiac surgery survival rate, which is more than 95% in highly developed institutions, and the incidence of severe neurological sequel have been dramatically improved over time, so that attention has been focused on long term functional morbidity, especially neurodevelopmental disabilities. Unfortunately, neurological injury still occurs in children as a consequence of heart surgery with cardiopulmonary bypass (CPB). In large USA centers, 40-50% of children aged 4-5 years who were submitted to complex heart surgeries during the neonatal period or before six months of age present delayed neurocognitive development, including cognitive, motor and emotional deficit, delayed fine motor skills, language, speech and motor visual organization, as well as attention deficit and hyperactivity^[1-3]^. Recently, in neonates undergoing cardiac surgery with CPB, who were monitored with cerebral oximetry (rScO_2_) with near-infrared spectroscopy technology (NIRS) and cerebral blood flow (CBF) with Doppler ultrasound, the swift treatment of ischemia-hypoxia detected events resulted in better neurodevelopmental outcomes accessed by Bayle Scale III for language and motor performance^[4]^.

Severe neurological sequeale (coma, cerebral palsy, choreoathetosis, epilepsy, and hemiparesis) are much less frequent (occurring in less than 10% of cases). The actual incidence of deficits is difficult to define and may only be evident several years after cardiac operation due to the limitations of functional evaluation of neonates and infants and to the potential changes produced by neuroplasticity. Because of the lifelong personal expenses and efforts associated with neurological sequels, timely investments in prevention and treatment are socially and individually justified. The major costs of prolonged intensive therapies in cases of serious neurological sequels should also be considered^[4-6]^.

Real-time neurologic and cardiorespiratory monitoring should be an integral part of neuroprotective strategies for pediatric patients requiring cardiac surgery. It also allows appropriate and swift management to detect and counteract decrease in cerebral oxygenation. Ideally, real-time monitoring should allow easy, reliable, and reproducible detection of adverse outcome events and their causes.

## MECHANISMS OF NEUROLOGICAL INJURY DURING HEART SURGERY

The etiology of brain damage is multifactorial regarding the time and mechanism of injury. One must consider that preoperative abnormalities (hyper/hypotonia, difficulty in feeding, choreoathetosis, spasticity, macro/microcephaly) are identified in 50% of neonates with congenital heart disease and are predictive factors of neurodevelopmental disability after heart surgery^[7]^. Magnetic resonance image (MRI) and computed tomography (CT) have demonstrated a 10-30% incidence in brain abnormalities such as metabolic dysfunction, structural anomalies and white matter damage (leukomalacia) in children with congenital heart disease. Leukomalacia has been identified in 50% of children submitted to heart surgery and is associated with diastolic hypotension and hypoxemia^[4,8]^.

The mechanisms of postoperative brain damage are still controversial, although hypoxemia, global ischemia and post ischemic reperfusion damage appear to be the major components. Other mechanisms include embolism, anesthetic neurotoxicity, inflammatory responses and modification of cell life-cycle development (apoptosis). The neurological damage of ischemia-hypoxia results from an inadequate oxygen and glucose supply needed to guarantee the energy requirements for the neurons, glia, endothelium, and support tissues that form the neurovascular unit^[4]^. The initial events of the ischemic-hypoxic process include excitotoxicity, intracellular calcium overload, energy failure (ATP depletion), oxidative and nitrosoactive stress that result in cell death (necrosis) in the infarction nucleus. Late events such as an inflammatory process and apoptosis (programmed death) are relevant regarding neuronal death in the penumbra tissue ischemia. All together, these phenomena are known as continuous necrosis-apoptosis of neonatal brain injury^[9]^. The degree of ischemia of the infarction nucleus is the primary risk factor that distinguishes ischemic damage from the penumbra region, as determined on animal models that evaluated the degree of hypoxia and cerebral blood flow^[10]^.

The reduction of neurological sequels by the handling of manageable risk factors is based on the interruption of the cerebral ischemia-hypoxia process as soon as it arises (within minutes of its occurrence) or by providing timely intervention while a therapeutic window still exists. This approach has received priority attention on the part of pediatricians, surgeons, and anesthesiologists. Manageable and modifiable intraoperative period factors include hematocrit during CPB, acid-base management, regional cerebral perfusion, cooling and rewarming during CPB rates, high cerebral vascular resistance, alteration in cerebral autoregulation (BP- rScO_2_ relationship) during deep hypothermia, glycemia, mean and diastolic BP, pulmonary vascular resistance, seizures, PaCO_2_, and cardiac output^[11-14]^. Brain oxygenation monitoring with NIRS or SsvcO_2_ (superior vena cava hemoglobin oxygen saturation), largely determined by cerebral venous flow, and real time functional integrity (electroencephalogram (EEG), evoked potentials) monitoring in real time are of primordial importance, and avoid or minimize the use of anesthetic associated with neurotoxicity (apoptosis) such as volatile anesthetics and ketamine should be part of advocated anesthesia cardiac pediatric surgery protocols^[4]^. Critical periods of cerebral oxygenation reduction typically occur during the induction of anesthesia when hypotension and decrease of cardiac output take place, cannulation for CPB, low-flow or variable flow during CPB, rewarming and separation from CPB^[15]^.

## EVALUATIONS OF BRAIN INJURY

The application of specific neurological monitoring for the detection of cerebral ischemia/hypoxia such as continuous brain oxygenation by the NIRS technique and transcranial Doppler or for immediate neurophysiological repercussions of neurological injury, processed EEG techniques such as BIS and evoked potentials may lead to rapid therapeutic intervention as well as refinement of risk reduction strategies. Prospective cohort study has shown that neurophysiological monitoring may afford a sound bases for rapid therapeutic intervention resulting in reduction of this complication from 26 to 6%^[9]^.

On the other hand, biochemical markers, neural band creatine kinase (CK-BB), neuron specific enolase (NSE) and S100β protein are also of great theoretical importance^[4]^ however, unfortunately, their determination still requires a too long time before a neuroprotective therapy can be started, in contrast to NIRS and functional neurophysiological evaluations can be performed in real time.

The growing attention devoted to the brain functioning development in children, particularly neonates, has been emphasizing the usefulness of neurological monitoring of cerebral hypoxia, blood perfusion anomalies and electrophysiological disorders acknowledge as a surrogate for neurologic injury events (seizures, delta pattern of the EEG)^[16]^. The most frequent employed brain monitors (neuromonitors) include NIRS (which measures regional oxygen saturation - rSO_2_), processed EEG and transcranial Doppler (which estimate cerebral blood flow-CBF). These monitors devices with other standard physiological monitoring techniques increase our ability to detect and prevent damage resulting from ischemia-hypoxemia, embolism, hypocapnia, arterial hypotension, low cardiac output, and hyperthermia during surgery and over postoperative intensive therapy. Functional tests of specific neural systems are seldom used as auditory evoked potentials (AEP) and somatosensory evoked potentials to assess the synaptic integrity of the neural pathways. Continuous measurements of arterial hemoglobin oxygen saturation (SaO_2_), systemic arterial pressure and central venous oxygen saturation (SvO_2_) do not predict in an adequate manner brain desaturation^[12,13]^. For this reason, continuous monitoring by cerebral NIRS is being adopted as a universal strategy (for all patients) for the detection of cerebral desaturation events that may occur in unpredictable manner, but that respond normally to therapeutic intervention^[6]^. Notwithstanding, the simultaneous measurement of cerebral and somatic rSO_2_ may provide a body map of cardiac output distribution, low cardiac output and/or specific low cerebral blood flow^[5]^.

## MEASUREMENT OF CEREBRAL OXYGENATION

The most frequently employed technique is the determination of SjO_2_ and rSO_2_ by NIRS, representing global (hemispheric) and regional oxygenation, respectively. The two variables are highly correlated and the tendencies are monitored^[17]^, both describing the relation between cerebral metabolic rate of oxygen (CMRO_2_) and oxygen supply. The NIRS has the advantage of not being invasive, of being a dynamic marker in real time, without requiring pulsatile method such as pulse oximetry, and being useful during CPB with hypothermia and during resuscitation after a cardiac arrest. The cerebral NIRS provides a reliable measure of venous saturation below the sensors, especially in small skulls such as those of neonates^[18]^. The cerebral regional oximetry (rScO_2_) is strongly correlated with neurological evolution in experiments of cerebral ischemia-hypoxia. In piglets submitted to progressive degrees of hypoxia, NIRS was strongly correlated with neurocognitive dysfunction and cerebral blood flow. Increased lactate production (measure of anaerobic metabolism), low frequency (<4 Hz) in the electrocardiogram, and energy failure (ATP depletion) occur when rSO_2_ fell to 44%, 42% and 33%, respectively. Changes in CBF are also correlated with cerebral rSO_2_ and with biochemical and electrophysiological markers. A 58% reduction of CBF marks the threshold for the installation of ischemia-hypoxia injury associated with a cerebral rSO_2_ of less than 50%^[19]^. During CPB, the period of rewarming carries the greatest risk for cerebral hypoxia regardless of appropriate SaO_2_^[15]^.

## FUNCTIONAL MEASUREMENTS OF BRAIN INJURY

The EEG during the per operatory period of pediatric heart surgery permits the detection of neocortical synaptic activities. The EEG pattern, in addition to cerebral perfusion, is influenced by the cerebral temperature, alert state, anesthetic drugs, and age. The interpretation of the EEG is operator-dependent and the signals may suffer electric interferences; however, the signals can be processed with various algorithms to reduce their complexity and the interpretation task. Fourier analysis of the EEG generates a series of numerical descriptors including spectral edge frequency, total potency and suppression ratio that may have a poorly consistent relationship with cerebral ischemia. The BIS quantitates the cortical activity by spatial non-coherence and has a probabilistic relation with the depth of anesthesia and the degree of sedation; however, it suffers the influence of various drugs and of temperature and of the supply of oxygen and glucose to the brain. At profound hypothermia, a body temperature of less than 28º C, the electrical activity may be processed to the isoelectric point, which may not be distinguished from profound anesthesia^[20]^. The relationship between cerebral perfusion and EEG abnormalities, although poorly consistent and nonlinear, may provide a clinically useful marker of cerebral ischemia (EEG frequency < 4 Hz, convulsive activities)^[21]^. However, when it is detected, the ischemic injury should have already occurred. Importantly, combined NIRS and EEG monitoring reduces the incidence of brain damage observed by magnetic resonance (an early marker)^[22]^.

On CPB, when usual clinical signs of anesthesia are not available, processed EEG technology monitoring, such as BIS, are advantageous anesthetic depth determination. BIS monitoring compute a number that range from zero (isoelectric) to the mean awake values in 90-100 range in adults, children and infants. BIS values are reported to decrease during hypothermia and anesthetic dose required to achieve BIS values of 40-60 (deep hypnosis levels) are decreased during hypothermia. During rewarming phase of hypothermia CPB in children, BIS is reported to increase, perhaps reflecting an actual increase of conscious levels. BIS monitoring is reported to help the detection central nervous system hypoperfusion and cerebral embolism. It has been combined with NIRS to detect cerebral ischemia during cardiac surgery in children^[22,23]^. During adult cardiac surgery, anesthesia controlled by BIS monitoring may facilitate titration of anesthetic agents, and decrease the hemodynamics disturbances. Inversely, fewer intraoperative hemodynamics events occur with BIS monitoring^[24]^. Additionally, BIS can predict bad neurological outcome in comatose survivors after cardiac arrest and induced hypothermia^[25]^.

Nowadays, NIRS is the most frequently used brain monitor due to practical reasons. The use of various continuous monitoring modalities such as regional oximetry (NIRS) and processed EEG (BIS), Doppler ultrasound are complementary rather than exclusive. Application of combination of these monitors might be the best current approach. Indeed, several authors have developed prevention measures and treatments algorithms based on multimodal neurological monitoring. However, evoked potentials, such as AEP, can be an additional tool to provide additional neurophysiological information regarding the functional integrity of the neuronal pathway on real time^[22]^.

## NEUROPROTECTIVE ANESTHESIA AND INTRAOPERATIVE IN-TENSIVE MEDICAL CARE

Neuroprotective strategies can be classified as preventive, reactive and reparative. Anesthesia and hypothermia are most involved with preventive therapeutics strategies since it is given in advance of the anticipated cerebral insult such as circulatory arrest, cumulating of periods of low-flow CPB, hypotension, cerebral hyperthermia and vasoconstriction during rewarming on CPB^[4]^. Reaction strategies requires cerebral oxygenation monitoring with NIRS or SsvcO_2_, combined with hemodynamic, arterial oxygenation, core body temperature and metabolic (PaCO_2_, glycemia, lactate) monitoring. Neurophysiological monitoring such as processed EEG (BIS) and AEP, and cerebral blood flow measurement with Doppler can be complementary and improve the fast detection and management of cerebral hypoxia.

Erythropoietin is the only available drug that has scientific rationale and pre-clinical data for two phases of neuroprotective strategies (preventive and reparative) and had safety and efficacy studies (Phase I/II) in neonate^[26,27]^. Nevertheless, despite of its extreme safety in neonate, future studies concerning its efficacy in cardiac surgery with CPB are required. Besides volatile anesthetics (sevoflurane, isoflurane), intravenous anesthetics GABAA agonist (propofol, midazolan) and NMDA antagonist (ketamine), that are routinely used during cardiac pediatric anesthesia as a complement of the high induction of opioid anesthesia, other adjuvant drugs can also be used because of their hypnotic, analgesic, vasodilator (sympatholytic effect), antiarrhythmic, anti-inflammatory effects and anticonvulsant effects. Among them, dexmedetomidine^[28-32]^, magnesium sulfate^[33-35]^, lidocaine^[36-38]^, chlorpromazine^[39]^, although presenting neuroprotective properties in animal model of ischemia/hypoxia, suffer from conflicting clinical evidence for their putative benefits. The clinical evidence for their benefits are still conflicting^[40-42]^. Dexmedetomidine has been used in combination with opioid in order to provide hypnosis and deep intraoperative analgesia during pediatric cardiac surgery^[30]^ and it is considered a therapeutic agent against ventricular and supraventricular tachyarrhythmias^[43]^. Chlorpromazine is an effective systemic vasodilator in neonate on CPB^[44]^. Interestingly, spontaneous moderate hypothermia (33-34°C) and permissive hypercapnia may contribute to organ protection during pediatric cardiac surgery with CPB^[44,45]^. The combination of dexmedetomidine, magnesium sulfate, lidocaine infusion and ketamine, called opioid-free anesthesia, is an acceptable, safe and effective anesthesia techniques in adult patients^[46]^.

Despite of intensive research on the field of neuroprotection with drugs and procedures (moderate hypothermia, remote preconditioning), the development of reliable clinical neuroprotective therapy during pediatric cardiac surgery which individually or in appropriate combination that can maintain blood pressure, cardiac output, and cerebral oxygenation, block the transmembrane and large volume shift, inflammatory, oxidative pathway, and the release of excitotoxic neurotransmitter (glutamate) or block their postsynaptic effects that leads to neuronal death have not been stablished yet. The advocated neuroprotective regimens have most of their evidence based on animal basic studies and translated to clinical practice - "bench-to-bedside approach". Proper animal's studies that reproduces the clinical conditions and assessment, validation and pilot studies to prove the concept in patients, particularly in neonate, followed by controlled studies are indicated. Recent dating regarding neuroprotection strategies may be controversy because there are multiple factors that contribute to brain injury and assessment of long-term neurocognitive outcome is problematic^[11,46,47]^.

Besides anesthetics and adjunct drugs above mentioned, other well accepted procedures and physiological controls are routinely applied during cardiac surgery with CPB, including mild to moderate hypothermia (core temperature of 33-34°C), deep hypothermia (core temperature of 17-26°C), hematocrit over 24%, glycemia >100 mg%, pH management, prophylactic anti-inflammatory, selective cerebral perfusion, ultrafiltration. A protocol for physiologic targets is established in order to guarantee appropriate cerebral and systemic oxygen supply: 1- MABP (mean arterial blood pressure) 40-50 and DBP (diastolic blood pressure) > 30 mmHg; 2- SsvcO_2_ or rScO_2_ >65% and > 75% during CPB; 3- arterial pulse profile visually normal; 4- mild hypothermia (33-34°C) before CPB and normothermia (35-36°C) after CPB; 5- glycemia = 100 - 180 mg%; 6- Htc > 35% and >25% (during CPB); 7- plasma calcium > 1.20 mmol/L; 8- plasma lactate < 2 mmol/L, 9- diuresis ≥ 1 ml/kg/h; 10- CVP = 6-10 mmHg, 11- SpO_2_ =80 - 95%; 12- PaCO_2_ = 35-55 mmHg; and 13- BIS = 45-70 in children over 6 months and core temperature over 30°C. These required end-points can almost always be accomplished through management of hemodynamic (volume loading and vasoactive drugs), ventilation patterns and FiO_2_, and anesthesia deepness through opioid dosing and adjuvant drugs. Specific cerebral ischemia-hypoxia can occur with low PaCO_2_ (<30 mmHg), high core temperature (>38˚C), and light anesthesia (pain, awareness). On the other hand, when cardiac output is critically low, permissive hypercapnia can lead to splanchnic and renal ischemia^[11,13]^.

Sedation for transport and monitoring can be promoted by midazolan (50-100 µg/kg) plus ketamine (0.5-1 mg/kg) boluses. An anesthetic induction that allows tracheal intubation, arterial and central venous cannulation can be carried out with midazolam (0.1mg/kg), lidocaine (1.5 mg/kg), fentanyl (5 µg/kg), and vecuronium (0.4 mg/kg). The anesthesia is maintained with fentanyl (50 µg/kg before CPB and more 25-150 µg/kg until the end of surgery), lidocaine (1.5 mg/kg/h), and dexmedetomidine infusion (1 µg/kg/h) started soon after arterial and central venous cannulation and physiologic and laboratorial monitoring. Magnesium sulfate (50 mg/kg followed by 15 mg/kg/h) is administered at the beginning of CPB. An infusion of norepinephrine (0.03-0.06 µg/kg/min) or boluses of vasopressors phenylephrine or ephedrine should be given to minimize the blood pressure and heart rate decrease caused by fentanyl, dexmedetomidine and magnesium administration, particularly those hemodynamic unstable or in critical condition. Recently, we changed the anesthetic technique by given continuous infusion of dexmedetomidine, magnesium sulfate and lidocaine without bolus, to avoid decrease in BP, HR and cardiac output (SvcsO_2_ or rScO_2_< 60%). Sevoflurane can be used for induction in children with no venous access and for short-time control of hyper dynamic responses (increases in systolic arterial pressure and heart rate above 30% of control levels). All the patients receive dexchlorpheniramine (0.2 mg/kg), hydrocortisone (20 mg/kg) and ranitidine (2 mg/kg). Chlorpromazine (0.5-5 mg/kg) is administered for arterial hypertension control. All patients receive milrinone (0.5-0.7 µg/kg/min), when starting the rewarming during CPB.

This anesthetic protocol is supported by basic studies and recent cohort that have demonstrated that high doses of fentanyl and benzodiazepine are associated with neuroprotection and less apoptosis, and higher cognitive scores and less new MRI brain injury while exposure to volatile anesthetics and ketamine lower the cognitive scores in neonate undergoing cardiac surgery^[28]^. There are, also, a strong body of evidences that dexmedetomidine can in fact be neuroprotective, anti-apoptotic, and antagonize the behavioral impairment caused by inhaled anesthetics in neonate^[28,29]^. The safety of general anesthetic in neonate has come under contention after accumulating reports from preclinical studies revealed that general anesthetic exposure leads to widespread apoptosis neurodegenerative and long-lasting cognitive and learning impairment in immature and developing brain (neonate). Animal studies and some clinical studies has shown the neurotoxicity of prolonged (more than 3 hours) or repeated exposure to GABAA agents (inhaled anesthetics, benzodiazepines, nitrous oxide, propofol, barbiturates) and NMDA receptors antagonists (ketamine) produce cognitive and behavioral deficits such as learning and memories deficits^[48]^. The ghost of the routinely anesthetic agents as a contributing factor for neuronal apoptosis and consequent neurocognitive impairment in human neonate remain a current concern. We believe that high induction dose of opioid anesthesia associated with alpha_2_ agonists (clonidine, dexmedetomidine) is a better alternative to current volatile anesthesia technique in neonate. On the other hand, the cardiovascular limitation of the developing heart to depressant effects of general anesthetic due to its limited contractility, low compliance and frequency dependency, significant hypotension and reduction of cardiac output associated with anesthesia can cause decrease in cerebral blood flow and oxygenation which can exaggerate drug neurotoxicity. Ketamine anesthesia induction can maintain blood pressure and heart rate, and despite of potential neurotoxicity of prolonged administration, a single dose of 0.5-1 mg/kg can, in fact, be neuroprotective^[49]^. Also, a short period of sevoflurane anesthesia to provide hypnosis and hemodynamic control, should not cause neurologic harm^[50]^.

The present advocated neuroprotective anesthetic regimen was used in 52 pediatric patients undergoing cardiac surgery with CPB. SsvcO_2_> 65% was considered the end-point criteria for hemodynamic and respiratory management, weaning from the CPB and to pediatric intensive care unit admission. In this retrospective cohort, with pediatric patients aging from 1 day to 14 years weighting 2-28 kg, SsvcO_2_ at the end of surgery varied from 67 to 87 % (76±6). One patient died at the end of the surgery and one had low SsvcO_2_<50% due to cardiac arrhythmia that improved after careful adjustment of the pacemaker. The fentanyl dose range was 56 to 170 µg/kg. Twenty-four percent of the patients received chlorpromazine (0.5-5 mg/kg), to control systemic hypertension during and after CPB. All patients were transported to PICU on milrinone (0.5-0.7 µg/kg/min) but less than 50% required catecholamine at the end of surgery. Although the perioperative clinical results yet observed seems very promising and warrant a clinical research trial to test organ protection efficacy, we must avoid over-enthusiasm in adoption this anesthetic technique for all patients with congenital heart disease undergoing undergoing cardiac surgery with CPB, and we should maintain healthy skepticism until controlled study are carried out.

Recently, we have been using a combined infusion of dexmedetomidine (1 µg/kg/h), magnesium sulfate (30 mg/kg/h), and lidocaine (2 mg/kg/h) associated with fentanyl (≥ 75 µg/kg). The infusion starts soon after arterial cannulation and is halved after one hour. All patients are monitored with cerebral and somatic (renal) regional oximetry and BIS (over 3 month of age). Various degree of systemic vasodilation at the end of surgery is a common feature. A protocol for study with a controlled, randomized, double-blind and stratified design was started, comparing the proposal anesthetic neuroprotective regimen against the standard anesthesia care with opioid-midazolan anesthesia. The primary outcome will be cerebral/somatic oximetry with NIRS technology, neurophysiological and biochemical organs injury markers, perfusion pressure and vasoactive drugs requirement, inflammatory markers (protein C reactive and IL-6), ventilation patterns FiO_2_, PEEP), organs perfusion pressures, oxygen extraction, duration of mechanical ventilation and length of stay in pediatric intensive care unit.

**Table:** 

Authors' roles & responsibilities	
JGK	Substantial contributions to the conception or design of the work; or the acquisition, analysis, or interpretation of data for the work; final approval of the version to be published
WVAV	Conception and design of the target clinical management criteria, responsible surgeon, critical review and manuscript drafting; final approval of the version to be published
LVG	Data acquisition and interpretation; final approval of the version to be published
FC	Design of the clinical target clinical managements, data interpretation, postoperative care; final approval of the version to be published
JA	Data acquisition and interpretation; final approval of the version to be published
ACM	Final approval of the version to be published
PHM	Preoperative care, pediatric patient's selection, data interpretation; final approval of the version to be published
